# Twice daily feeding during gestation improves maternal body reserves and fetal growth in sows under commercial conditions

**DOI:** 10.1093/tas/txag049

**Published:** 2026-04-29

**Authors:** Emmanuel O Aidelomon, Pavel Nevrkla, Naděžda Kernerová, Petr Tejml, Mohammed S Iliyasu, Emeka M Nnando

**Affiliations:** Department of Animal Husbandry Sciences, Faculty of Agriculture and Technology, University of South Bohemia in České Budějovice, Studentská 1668, České Budějovice, 370 05, Czech Republic; Department of Animal Breeding, Faculty of AgriSciences, Mendel University in Brno, Zemědělská 1, Brno, 613 00, Czech Republic; Department of Animal Husbandry Sciences, Faculty of Agriculture and Technology, University of South Bohemia in České Budějovice, Studentská 1668, České Budějovice, 370 05, Czech Republic; Department of Animal Husbandry Sciences, Faculty of Agriculture and Technology, University of South Bohemia in České Budějovice, Studentská 1668, České Budějovice, 370 05, Czech Republic; Department of Animal Husbandry Sciences, Faculty of Agriculture and Technology, University of South Bohemia in České Budějovice, Studentská 1668, České Budějovice, 370 05, Czech Republic; Department of Technology and Cybernetics, Faculty of Agriculture and Technology, University of South Bohemia in České Budějovice, Studentská 1668, České Budějovice, 370 05, Czech Republic

**Keywords:** backfat thickness, body weight, feeding frequency, fetal growth, gestation, parity

## Abstract

The aim of this study was to investigate whether splitting the daily feed allowance into two meals could improve maternal condition and fetal growth. A total of 338 Landrace × Yorkshire (Danbred) sows of parities 1–9 were used. Sows were first grouped by parity into three blocks (parity 1–2, *n* = 129; parity 3–5, *n* = 154; parity ≥ 6, *n* = 55). Within each block, animals were randomly assigned to one of two feeding frequency treatments: once daily (*n* = 156; 08:00 h) or twice daily (*n* = 182; 08:00 and 16:00 h). All sows received identical gestation diets across different stages (3.0, 2.5, and 3.3 kg during early, mid, and late gestation, respectively). The diet provided 3.25 Mcal ME/kg (12.9% crude protein), resulting in daily ME intakes of 9.75, 8.13, and 10.73 Mcal during early (days 1–35), mid (days 36–80), and late (days 81–110) gestation, respectively. Body weight (BW) and backfat thickness (BF) were measured on days 1, 35, 80, and 110 of gestation. Data were collected on piglet birth weight, farrowing duration, birth weight coefficient of variation (CV), number of live-born piglets, stillborn piglets, and mummified fetuses. Average daily feed intake (ADFI) did not differ between groups. Sows fed twice daily had significantly higher BW from mid-gestation onward (days 35, 80, and 110) and achieved greater cumulative weight gain (26.5 kg vs. 22.4 kg; *P* < 0.001). Backfat gain was higher in sows fed twice daily. Piglets from sows fed twice daily had significantly higher mean birth weights (1.31 kg vs. 1.17 kg; *P* < 0.001) accompanied by a reduced proportion of low-birth-weight piglets (<1.10 kg). Twice daily feeding did not affect litter size, farrowing duration, or birth weight CV, whereas parity significantly affected litter size and birth weight CV. There was no interaction between feeding frequency and parity. In conclusion, the present study shows that increasing feeding frequency to twice daily during gestation improves maternal body reserves and fetal growth in sows. This feeding approach offers a simple, practical management strategy to alleviate metabolic pressure and improve productivity in modern swine farming.

## Teaser

Feeding sows twice daily during gestation is a practical, low-cost strategy to improve maternal body reserves and increase piglet birth weights without increasing total feed intake. This approach helps reduce metabolic pressure on the sow and results in heavier, more viable piglets, potentially enhancing overall herd productivity.

## Introduction

Progress in selection has produced hyper-prolific sows, with increases in the average number of piglets born per sow and larger litter sizes (Knap 2023; Oliviero, 2023), contributing to improved production efficiency in pig farming systems (Oliveira et al. 2020). However, this increase is accompanied by considerable physiological challenges during late gestation, when fetal nutritional demands rapidly increase, accounting for approximately 70%–80% of fetal growth (Mosnier et al. 2010). Sows undergo critical metabolic adaptations to meet this demand, such as progressive insulin resistance, which prioritizes glucose allocation to fetuses while putting sows at risk of excessive mobilization of body reserves, impaired lactation, and subsequent reproductive performance (Mosnier et al. 2010; Cheng et al. 2019). This challenge may be more pronounced in multiparous sows due to repeated metabolic and oxidative stress associated with successive reproductive cycles (Cozannet et al. 2018; Zhao and Kim 2020). Another consequence of selection for increased litter size is a decline in piglet birth weight, likely due to increased competition for available nutrients within the uterine environment (van den Bosch et al. 2023; Ruggeri et al. 2025), coupled with greater variation in birth and weaning weight, which is associated with increased mortality risk during the lactation period (Feldpausch et al. 2019; Buthelezi et al. 2024). Smaller piglets face higher mortality rates, reduced growth rates, and poorer market performance (Mallmann et al. 2019), while heavier littermates tend to have greater access to colostrum, further disadvantaging low-birth-weight piglets (Vodolazska et al. 2023).

Nutritional strategies play a major role in mitigating these physiological and developmental difficulties during gestation. Most research has focused on optimizing nutrient composition and dietary supplementation strategies (Monteiro et al. 2025; Wang et al. 2025). In contrast, feeding frequency has received comparatively less attention, although it has been shown to influence behavioral and physiological responses in sows (Manu et al. 2020). When sows are fed once daily, prolonged fasting intervals between meals may alter physiological responses, influence behavioral patterns and stress-related endocrine responses, including cortisol secretion, in gestating sows (Manu et al. 2020). These effects may be particularly relevant during late gestation, when insulin resistance is already present, and nutrient requirements are high (Gerritsen et al. 2010). Consequently, such metabolic and physiological challenges are likely to place greater strain on sows, particularly hyper-prolific, multiparous sows (Cheng et al. 2019).

Therefore, the objective of this study was to evaluate the effects of feeding frequency during gestation on sow body condition, reproductive performance, and piglet birth weight across parity groups, to optimize both sow and piglet performance. We hypothesized that feeding sows twice daily during gestation would improve maternal body reserves and enhance piglet birth weight.

## Materials and methods

### Location, animals, and experimental design

This experiment was conducted on a commercial farm located in Strakonice, South Bohemia Region (49°16′N, 13°54′E), Czech Republic, between January and June 2024. During the study period, the average, minimum, and maximum regional temperatures were approximately 4.6 °C, −14.4 °C, and 17.6 °C, respectively, with a relative humidity of 85.3%. A total of 338 Landrace × Yorkshire (Danbred) sows of parities 1–9 were used in the study. Sows were stratified by parity into three blocks (parity 1–2, *n* = 129; parity 3–5, *n* = 154; parity ≥ 6, *n* = 55). Within each block, animals were randomly allocated to one of two feeding frequency treatments: once daily feeding (*n* = 156; 08:00 h) or twice daily feeding (*n* = 182; 08:00 and 16:00 h). All procedures were approved by the University of South Bohemia Institutional Animal Scientific Research Committee and complied with the EU Directive on the Protection of Animals Used for Scientific Purposes.

### Housing, management, and feeding regimens

Following artificial insemination with fresh diluted semen from Duroc boars, pregnancy was confirmed using an ultrasound pregnancy detection device at 4 weeks after insemination. From insemination to 4 weeks post-insemination, sows were housed individually in breeding stalls (211 cm in length and 64 cm in width). Sows confirmed pregnant were moved to a group housing facility with electronic sow feeders (ESF) under controlled environmental conditions (22 ± 1 °C; 60%–70% humidity). Each pen housed approximately 42–45 sows, providing a total pen area of 100.8–108.0 m^2^, corresponding to approximately 2.4 m^2^ per sow. Five days before farrowing, sows were moved to individual farrowing pens measuring 2.0 × 2.4 m (4.8 m^2^), equipped with farrowing crates (2.0 × 0.65 m; 1.3 m^2^) and creep areas with heat lamps and rubber mats that provided a warm resting area for piglets. Within 24 h of birth, the umbilical cords of piglets were cut and disinfected. Teeth clipping and tail docking were carried out at 1 day of age. Male piglets were surgically castrated during the first week of life. All piglets received an intramuscular iron injection. Cross-fostering was performed within 48 h after farrowing to balance litter size according to the number of functional teats per sow. Health management and vaccination were overseen by a veterinarian. Sows received identical standardized commercial gestation diets (Table 1), formulated to meet or exceed the NRC (2012) guidelines for gestating sows, providing 3.25 Mcal ME/kg with 12.9% crude protein. The daily feed allowance followed a phased regimen: 3.0 kg from days 1 to 35, 2.5 kg from days 36 to 80, and 3.3 kg from days 81 to 110, yielding daily ME intakes of 9.75, 8.13, and 10.73 Mcal/d during early, mid, and late gestation, respectively (Table 2). Water was provided ad libitum. After farrowing, sows were fed a commercial lactation diet (Table 1) containing 13.3% crude protein, 3.69 Mcal ME/kg with 0.8% lysine.

**Table 1:** Composition and nutrient analysis of gestation and lactation diets.

Ingredients (%)	Gestation diet	Lactation diet
**Wheat**	45.00	59.23
**Barley**	33.00	10.00
**Oats**	5.00	2.00
**Soybean meal**	13.90	19.50
**Vegetable oil**	0.00	5.00
**Calcium carbonate**	1.20	1.50
**Monocalcium phosphate**	0.70	1.00
**Sodium chloride**	0.40	0.40
**Premix^a^**	0.50	0.50
**l-Lysine**	0.20	0.55
**l-Threonine**	0.08	0.20
**dl-Methionine**	0.02	0.12
**Total**	100.00	100.00
**Analyzed composition**		
**Metabolizable energy Mcal/kg**	3.25	3.69
**Crude protein (%)**	12.9	13.3
**Crude fat (%)**	2.4	7.2
**Crude fiber (%)**	4.2	2.8
**SID Lysine (%)^b^**	0.55	0.80
**Calcium (%)**	0.85	0.90

a Premix content/Kg of Diet: Vitamin A, 12,500 IU; Vitamin D3, 2500 IU; Vitamin E, 40 mg; Vitamin K, 2.5 mg; vitamin B1, 3 mg; vitamin B2, 5.5 mg; niacin, 55 mg; calcium pantothenate, 11.5 mg; vitamin B6, 5 mg; vitamin B12, 25 µg.

b SID, Standardized ileal digestible.

**Table 2:** Energy intake by gestation phase.

Phase	Days	Duration (d)	Feed (kg/d)	Daily ME (Mcal/d)^a^	Phase ME (Mcal)^b^
**Early**	1–35	35	3.0	9.75	341.3
**Mid**	36–80	45	2.5	8.13	365.6
**Late**	81–110	30	3.3	10.73	321.8
**Total**	1–110	110	–	–	1028.7

a Daily ME = feed allowance (kg/d) × 3.25 Mcal/kg.

b Phases = measurement points in this study (d1–35, d36–80, d81–110).

### Data collection and measurements

The body condition of sows was monitored during gestation and at farrowing. The body weight (BW) and backfat thickness (BF) were recorded individually on days 1, 35, 80, and 110 of gestation. Sows were weighed individually using a calibrated digital scale before feeding, and BF was measured using an ultrasound device at the P2 site (approximately 6–7 cm from the midline at the last rib). These gestational stages were selected to represent key physiological phases: baseline (day 1), completion of the placentation process (day 35), onset of the rapid fetal growth phase (day 80), and a pre-farrowing assessment of energy reserves (day 110), modified from Manu et al. (2019a). Average daily feed intake (ADFI) and changes in BW and BF during gestation (days 1–110) were also calculated. Reproductive performance was recorded for each sow at farrowing. The total number of live-born piglets, stillborn piglets, and mummified fetuses was recorded at birth. Farrowing duration was calculated as the time of the last piglet birth minus the time of the first piglet birth. Piglet birth weight was recorded within 12 h of birth. The birth weight coefficient of variation (CV) was calculated. Three birth weight categories were defined. Piglets were classified as low (<1.10 kg), intermediate (1.10–1.39 kg), or high (≥1.40 kg) birth weight, with thresholds modified from Manu et al. (2019a).

### Statistical analysis

Data were analyzed using TIBCO Statistica (version 14.0; TIBCO Software Inc.). Normality and homogeneity of variance were assessed with the Shapiro–Wilk test and Levene’s test, respectively. The sow was used as the experimental unit for all analyses. A two-way factorial mixed model was used to evaluate the effects of feeding frequency (once daily vs. twice daily), parity group (Parity 1–2, Parity 3–5, Parity ≥ 6), and their interaction on sow performance, body condition, and reproductive traits. Feeding frequency, parity group, and their interaction were included as fixed effects. Repeated measures of sow BW and BF across gestation (days 1, 35, 80, and 110) were analyzed using a repeated measures mixed model, with time as the repeated factor and sow as the subject. An autoregressive covariance structure was selected based on the lowest Akaike information criterion (AIC). The ADFI, BW change, and BF change during gestation were analyzed using the same mixed model approach without repeated measures. Reproductive performance variables, including total born, live-born, stillborn, mummified fetuses, farrowing duration, piglet birth weight, and birth weight CV, were analyzed using mixed linear models with feeding frequency, parity group, and their interaction as fixed effects. Piglet birth weight was analyzed at the litter level using litter means. Data are presented as means ± standard deviation. Differences were considered significant at *P* < 0.05, and tendencies were discussed when 0.05 ≤ *P* < 0.10. The Tukey–Kramer test was used for multiple comparisons among means.

## Results

### Energy intake, feed intake, and body condition changes in sows

Total ME intake across gestation averaged 9.35 Mcal/d, with phase-specific daily intakes of 9.75 Mcal/d (days 1–35), 8.13 Mcal/d (days 36–80), and 10.73 Mcal/d (days 81–110). Cumulative gestational ME intake was 1,028.7 Mcal (Table 2). There was no effect of feeding frequency, parity, or their interaction on ADFI across all gestation phases (days 1–35, days 36–80, and days 81–110; *P* ≥ 0.287; Table 3). Sows fed twice daily had higher BW at day 35 (*P* = 0.034), day 80 (*P* = 0.012), and day 110 (*P* = 0.022; Table 3). Cumulative BW gain over gestation (days 1–110) was greater in sows fed twice daily (26.5 ± 7.8 kg) than in sows fed once daily (22.4 ± 8.1 kg; *P* < 0.001). Backfat thickness was higher in sows fed twice daily at day 80 (*P* = 0.014) and day 110 (*P* = 0.025), and BF gain over gestation was higher in sows fed twice daily (1.6 ± 1.4 mm) compared with sows fed once daily (0.9 ± 1.5 mm; *P* < 0.001). Parity affected BW at all measured time points (*P* < 0.001) and BF from day 35 onward (*P* ≤ 0.012; Table 3). No feeding frequency × parity interaction was observed for any body condition parameter (*P* ≥ 0.385). Detailed parity by treatment means for all measured variables are presented in Supplementary Table S1.

**Table 3:** Effect of feeding frequency and parity on sow body weight, backfat thickness and feed intake.

Parameters	Feeding frequency		Parity			*P*-value		
	Once	Twice	1–2	3–5	≥6	*F*	*P*	*F* × *P*
**Number of sows**	156	182	129	154	55			
**ADFI (kg/d)**								
** d 1–35**	2.71 ± 0.37^2^	2.69 ± 0.41	2.65 ± 0.39	2.73 ± 0.38	2.72 ± 0.42	0.723	0.401	0.588
** d 36–80**	2.27 ± 0.51	2.29 ± 0.49	2.24 ± 0.52	2.31 ± 0.48	2.28 ± 0.50	0.655	0.287	0.812
** d 81–110**	2.84 ± 0.62	2.86 ± 0.59	2.80 ± 0.61	2.88 ± 0.58	2.85 ± 0.63	0.814	0.335	0.745
**BW (kg)**								
** d 1**	192.9 ± 27.5	195.1 ± 26.8	185.4 ± 22.1^a-c^	198.3 ± 25.7^b^	205.8 ± 29.3^a^	0.285	<0.001	0.610
** d 35**	196.4 ± 20.1	201.2 ± 19.5	189.5 ± 18.3^b^	203.8 ± 17.9^a^	207.1 ± 21.6^a^	0.034	<0.001	0.422
** d 80**	205.4 ± 23.8	213.7 ± 24.1	197.2 ± 21.5^b^	216.5 ± 22.3^a^	219.3 ± 25.0^a^	0.012	<0.001	0.385
** d 110**	215.3 ± 26.3	221.3 ± 25.9	206.8 ± 23.4^b^	225.9 ± 24.1^a^	228.7 ± 27.5^a^	0.022	<0.001	0.451
** BW change (d 1–110, kg)**	22.4 ± 8.1	26.5 ± 7.8	21.4 ± 7.5^b^	27.6 ± 7.2^a^	22.9 ± 8.4^b^	<0.001	<0.001	0.205
**BF (mm)**								
** d 1**	15.3 ± 3.1	15.5 ± 3.1	14.8 ± 2.9	15.7 ± 3.0	15.9 ± 3.4	0.542	0.079	0.887
** d 35**	15.6 ± 2.8	15.9 ± 2.7	15.1 ± 2.5^b^	16.2 ± 2.6^a^	16.1 ± 3.0^a^	0.315	0.012	0.721
** d 80**	16.3 ± 2.5	17.1 ± 2.5	15.9 ± 2.3^b^	17.2 ± 2.4^a^	17.3 ± 2.7^a^	0.014	<0.001	0.534
** d 110**	16.2 ± 2.7	17.1 ± 2.7	15.8 ± 2.5^b^	17.2 ± 2.6^a^	17.3 ± 2.9^a^	0.025	<0.001	0.608
** BF change (d 1–110, mm)**	0.9 ± 1.5	1.6 ± 1.4	1.0 ± 1.3^b^	1.5 ± 1.4^a^	1.4 ± 1.6^a^	<0.001	0.015	0.837

a-c Values are presented as mean ± SD.

2 Means within a row with different superscript letters differ (*P* < 0.05; Tukey’s test).

### Piglet birth weight and sow reproductive performance

Feeding frequency did not affect total born (*P* = 0.551), live-born (*P* = 0.409), stillborn (*P* = 0.960), or mummified fetuses (*P* = 0.575). Parity influenced total born, live-born, and stillborn piglets (*P* < 0.001), with the greatest values observed in parity 3–5 sows. No feeding frequency × parity interaction was observed for any reproductive trait (*P* ≥ 0.134). Piglets born to sows fed twice daily had higher mean birth weight (1.31 ± 0.21 kg) than those from sows fed once daily (1.17 ± 0.14 kg; *P* < 0.001), whereas parity had no effect on mean birth weight (*P* = 0.306), and no feeding frequency × parity interaction was observed (*P* = 0.455). Birth weight CV was not affected by feeding frequency (*P* = 0.731) but was influenced by parity (*P* = 0.015). There was a tendency for longer farrowing duration in sows fed twice daily compared with sows fed once daily (288 ± 105 vs. 256 ± 99 min; *P* = 0.054), whereas parity and the feeding frequency × parity interaction had no effect (Table 4).

**Table 4:** Effect of feeding frequency and parity on piglet birth weight and sow reproductive performance.

Parameters	Feeding frequency		Parity			*P*-value		
	Once	Twice	1–2	3–5	≥6	*F*	*P*	*F* × *P*
**Sow number**	156	182	129	154	55			
**Total born**	21.1 ± 4.5^1^	21.4 ± 5.1	19.3 ± 5.4^c^	22.8 ± 4.3^a^	21.8 ± 4.2^b^	0.551	<0.001	0.305
**Live-born**	19.0 ± 4.0	19.4 ± 4.5	17.8 ± 4.7^c^	20.3 ± 3.8^a^	19.6 ± 3.8^b^	0.409	<0.001	0.270
**Stillborn**	1.4 ± 1.7	1.5 ± 2.0	1.0 ± 1.9^b^	1.8 ± 1.8^a^	1.7 ± 1.8^a^	0.960	<0.001	0.716
**Mummies**	0.7 ± 1.2	0.5 ± 0.8	0.5 ± 1.2	0.7 ± 0.9	0.5 ± 0.7	0.575	0.079	0.134
**Piglet birth weight (kg)**	1.17 ± 0.14	1.31 ± 0.21	1.22 ± 0.20	1.23 ± 0.20	1.25 ± 0.18	<0.001	0.306	0.455
**Birthweight CV(%)**	21.3 ± 4.1	20.6 ± 3.8	20.0 ± 3.9^b^	21.5 ± 3.7^a^	21.9 ± 4.3^a^	0.731	0.015	0.588
**Farrowing duration (min)**	256 ± 99	288 ± 105	275 ± 101	263 ± 100	272 ± 104	0.054	0.812	0.421

1 Values are presented as mean ± SD.

2 Means within a row with different superscript letters differ (*P* < 0.05; Tukey’s test).

## Discussion

Selecting sows for increased litter size has improved swine production efficiency; however, it has also introduced physiological and nutritional challenges, particularly during late gestation (Theil et al. 2023; Cantin et al. 2025). Feeding sows once daily prior to farrowing may exacerbate metabolic and productive challenges, as less frequent feeding has been associated with greater postprandial fluctuations in glucose and insulin concentrations (Zięcik et al. 2002), and a single daily meal prior to farrowing has been linked to greater sow body condition losses, higher preweaning mortality, and reduced piglet weaning weight compared with more frequent or ad libitum feeding strategies (Gourley et al. 2020). Therefore, increasing feeding frequency has been proposed as a management strategy to improve metabolic regulation and overall reproductive performance. In the present study, feeding pregnant sows twice daily improved maternal body reserves and supported fetal growth, as evidenced by increased maternal BW, BF, and piglet birth weight under commercial conditions.

### Maternal body reserves in sows

Optimizing the physical condition of sows is crucial for improving their well-being. Our findings show that feeding gestating sows twice daily increases BW and BF, while ADFI remains unchanged. This suggests that the improvements were not driven by increased feed intake. Consistent with this, sows fed twice daily under controlled energy intake have been reported to show a tendency for increased BF gain during gestation (Manu et al. 2019b), indicating potential changes in energy partitioning. Additionally, feeding frequency has been shown to modify postprandial hormonal responses in other species, which may further influence nutrient utilization (Solomon et al. 2008). Such improved nutrient availability may be particularly relevant in hyper-prolific sows (Cantin et al. 2025), which have elevated nutrient demands to support rapid fetal growth, as evidenced by improved metabolic status, body condition, and reproductive outcomes under optimized feeding strategies. Changes in body reserves during gestation are closely associated with piglet birth weight and growth performance (Ha et al. 2024). Backfat thickness is a key indicator of sow nutritional status and plays an important role in predicting nutrient requirements and reproductive performance (Peng et al. 2025). From our results, sows fed twice daily had greater BW from mid-gestation onward (days 35–110), as well as greater total BW gain over gestation. This effect became more pronounced as gestation progressed. These findings highlight the importance of feeding frequency during late gestation, a critical period characterized by rapid fetal and mammary growth and increased nutrient demand (Theil et al. 2022; Huber 2025). Nutritional strategies during late gestation can influence nutrient partitioning between maternal reserves and conceptus development, thereby affecting piglet performance and sow condition (McClellan et al. 2025; Mehtab et al. 2026). Improved BW gain under twice daily feeding indicates reduced maternal tissue mobilization, which may contribute to enhanced reproductive sustainability in high-producing sows. These results align with those of Holt et al. (2006), who found that sow BW and BF were significantly higher in the twice daily fed group under equal feed allowance. However, our results differ from those of Jung et al. (2024), who reported that sows fed once daily tended to gain more weight during gestation than those fed twice daily. These differences may be attributed to sow prolificacy and associated physiological demands, as our study used sows with very large litters.

With respect to parity, our results showed clear differences in sow BW and BF across gestation, with higher-parity sows (≥3) exhibiting greater values than younger sows. These findings are consistent with previous reports demonstrating parity-related differences in body reserve dynamics and responses to nutrient intake, where younger females generally have lower overall BW and BF and may mobilize more tissue despite showing comparable or even greater BW gain during specific phases of gestation (Gonçalves et al. 2016; Manu et al. 2019b). Notably, no interaction between feeding frequency and parity was observed, indicating that feeding twice daily benefited animals across all parity groups, including higher-parity sows that may be more susceptible to excessive body condition loss under commercial conditions. These findings are particularly relevant for modern commercial herds. Consistent with the absence of a feeding frequency × parity interaction, the parity by treatment breakdown (Supplementary Table S1) showed similar responses across parity groups.

The daily ME intakes observed in the present study (9.75, 8.13, and 10.73 Mcal/d for early, mid, and late gestation, respectively) were higher than the values predicted by the NRC (2012) model (approximately 6.5 Mcal/d in early to mid-gestation and 7.5–7.7 Mcal/d in late gestation). Similarly, ME intakes in the present study exceeded values reported by Manu et al. (2019b) across all gestation stages (6.92, 7.13, and 7.40 Mcal/d for early, mid, and late gestation, respectively), with the greatest differences observed in early (+2.83 Mcal/d) and late gestation (+3.33 Mcal/d). These results indicate that sows in the present study were supplied with energy above established requirements. Notably, the total gestational ME intake (1028.7 Mcal) was identical between treatment groups, as both received the same feed allowances, indicating that the observed differences in maternal body reserves and piglet birth weight between treatments were attributable to the meal distribution pattern rather than total energy supply. Collectively, these findings highlight the importance of meal frequency as a management strategy independent of overall energy intake.

We acknowledge that a methodological limitation related to ESF systems in group housing should be considered when interpreting the feeding frequency contrast in this study. Although sows in the once-daily treatment were allocated their full daily ration in a single feeder access, it cannot be excluded that a small proportion of animals, particularly at higher feed allowances (3.0–3.3 kg/d), may have consumed their ration across multiple visits due to gut-fill limitations. However, studies on ESF systems indicate that group-housed gestating sows may visit the feeder multiple times per day, with a substantial proportion of these being non-feeding visits, reflecting behavioral interactions with the system rather than repeated feed consumption, while overall feed intake remains constrained by the programmed daily allocation rather than ad libitum feeding (Chapinal et al. 2008; Olsson et al. 2011; Vargovic et al. 2021). This limitation was negligible during mid-gestation when feed allowance was lower (2.5 kg/d). If some control sows did consume their ration over multiple visits, the effective contrast in feeding frequency between treatments would have been reduced, representing a conservative bias. Therefore, the observed improvements in BW gain, BF, and piglet birth weight in sows fed twice daily may underestimate the true effect of feeding frequency under strictly controlled single-meal conditions.

### Fetal growth and piglet birth weight

Improving fetal growth in prolific sows remains a major challenge in modern swine production, as selection for increased litter size intensifies intrauterine crowding and nutrient competition, thereby reducing piglet birth weight (Quiniou et al. 2002; Richard 2013; Tucker et al. 2022). Our study shows that feeding sows twice daily during gestation significantly increased piglet birth weight, demonstrating fetal growth can be enhanced through nutritional management strategies that optimize maternal body condition and nutrient availability. This finding complements previous studies by Amdi et al. (2013) and Ren et al. (2017), who reported that maternal body reserves and gestation feeding levels play key roles in determining piglet birth weight and early development. In addition, physiological and behavioral factors, including potential changes in nutrient utilization and stress responses, may also contribute to the observed outcomes; however, these mechanisms were not directly evaluated in the present study and require further investigation. In contrast, our results differ from those reported by Jung et al. (2024) and Manu et al. (2019b), who found that altering feeding frequency during gestation had no significant effect on piglet birth weight or overall litter performance. The discrepancies between studies may be attributed to interactions among genetic, environmental, and management practices. These may include differences in genetic lines, feed quality, sow physiological status, and housing or management practices, all of which can independently influence sow nutrition and fetal development. Although feeding frequency has been proposed as a management strategy to improve sow performance, current evidence indicates that its effects on piglet birth weight and litter performance are inconsistent across studies.

Previous studies have demonstrated that increasing feed allowance during late gestation, particularly in bump-feeding strategies, can enhance sow BW gain and BF deposition without consistently improving piglet birth weight. For example, increasing feed intake beyond maintenance requirements during late gestation had no effect on piglet birth weight or litter performance despite greater maternal energy intake (Liu et al. 2020), indicating that increased nutrient supply alone does not necessarily translate into enhanced fetal growth. Similarly, increasing feeding levels from day 90 of gestation resulted in greater BW gain and body condition without significant improvements in piglet birth weight or litter traits (Mallmann et al. 2019), supporting the concept that maternal tissue deposition may be prioritized over fetal development under certain conditions. However, other studies have reported positive effects of increased feeding allowance on piglet birth weight and lactation performance (Ampode et al. 2023), suggesting that responses to increased feed intake may vary depending on parity, experimental conditions, and management practices. In addition, nutritional strategies during gestation are critical for sow performance and longevity (Monteiro et al. 2025), further emphasizing the importance of optimizing feeding approaches rather than simply increasing feed allowance. In contrast to these studies, the present results showed that sows fed twice daily had greater BW gain (26.5 vs. 22.4 kg), BF deposition (1.6 vs. 0.9 mm), and higher piglet birth weight (1.31 vs. 1.17 kg) despite similar daily feed intake across treatments. This indicates that the observed improvements were not driven by increased energy intake but rather suggested enhanced energetic efficiency and nutrient partitioning towards both maternal tissue and fetal growth.

Feeding frequency did not influence the number of live-born piglets, stillborn piglets, or mummified fetuses in the present study. This agrees with previous findings (Wittmann 1986; Holt et al. 2006; Jung et al. 2024), indicating that feeding frequency does not influence overall litter performance. Parity significantly affected litter size and birth weight CV in the present study. The increase in litter size with advancing parity suggests improved reproductive capacity, potentially due to higher ovulation rates and enhanced physiological maturity of older sows. Similar findings have been reported, where multiparous sows, particularly those in mid-parity (3–5), produce larger litters compared to younger sows (Buthelezi et al. 2024).

We used the NRC (2012) gestation model to estimate the additional ME required by increased feed supply alone to replicate the observed improvements in maternal tissue deposition and fetal growth in sows fed twice daily. For maternal tissue deposition, the observed treatment differences (+4.1 kg BW gain and + 0.7 mm BF) were converted to protein and lipid mass using NRC (2012) body composition assumptions (protein: 0.160 kg/kg BW gain) and established relationships between backfat thickness and lipid deposition (Dourmad et al. 1997), assuming approximately 0.9 kg lipid per mm BF. Based on this assumption, the observed increase of 0.7 mm BF corresponds to approximately 0.63 kg of additional lipid deposition. The ME required for tissue deposition was calculated using efficiency coefficients (*k*ₚ = 0.54 for protein; *k*ₗ = 0.74 for lipid) and tissue energy densities (5.686 Mcal/kg protein; 9.456 Mcal/kg lipid), yielding an estimated requirement of approximately 15.0 Mcal. For fetal growth, the observed increase in piglet birth weight (+0.14 kg/piglet across an average of 19.2 live-born piglets) represents an extra 2.69 kg of litter mass. Using the NRC (2012) energy density for neonatal piglet tissue (approximately 2.12 Mcal/kg) and the efficiency of ME use for conceptus growth (*k*ᶜ = 0.61), the ME required to produce this additional litter mass was estimated at approximately 9.3 Mcal. In total, the combined ME required to support both maternal and fetal responses was approximately 24.3 Mcal. The magnitude of the response observed in the present study suggests a potential role of feeding frequency in improving the efficiency of energy utilization.

## Conclusion

Increasing feeding frequency to twice daily during gestation improved sow body reserves and increased piglet birth weight without affecting total feed intake. Litter size, stillbirths, and mummified fetuses, remained unchanged. These effects were consistent across parity groups. The findings suggest that increasing feeding frequency during gestation may enhance sow condition and support improved fetal development, representing a feasible management strategy in practice. However, the absence of direct metabolic measurements limits the interpretation of underlying physiological mechanisms, and the study was conducted within a single production system. In addition, piglet performance was only evaluated at birth. Future research should incorporate metabolic indicators and assess long-term outcomes, including piglet survival, growth performance, and lifetime productivity, across diverse genetic lines and management systems. Overall, increasing feeding frequency during gestation appears to be an effective approach to improve sow body condition and piglet birth weight.

## Supplementary Material

txag049_Supplementary_Data

## Abbreviations

ADFI: average daily feed intake
BF: backfat thickness
BW: body weight
CV: coefficient of variation
ESF: electronic sow feeder

## References

[txag049-B1] Amdi C. , Giblin L., Hennessy A. A., Ryan T., Stanton C., Stickland N. C., Lawlor P. G. 2013. Feed allowance and maternal backfat levels during gestation influence maternal cortisol levels, milk fat composition and offspring growth. J. Nutr. Sci. 2:e1. 10.1017/jns.2012.20 25191557 PMC4153285

[txag049-B2] Ampode K. M. B. et al 2023. Bump feeding improves sow reproductive performance, milk yield, piglet birth weight, and farrowing behavior. Animals. 13:3148. 10.3390/ani13193148 37835754 PMC10571924

[txag049-B3] Buthelezi N. L. et al 2024. Effects of parity, season of birth, and sex on within-litter variation and pre-weaning performance of F1 large white × landrace pigs. Vet. World. 17:1459–1468. 10.14202/vetworld.2024.1459-1468 39185040 PMC11344108

[txag049-B4] Cantin J et al 2025. Optimizing sow and litter performance via a comprehensive service-to-weaning feeding regimen. Animals. 15:2821. 10.3390/ani15192821 41096416 PMC12523336

[txag049-B5] Chapinal N et al 2008. Feeder use patterns in group-housed pregnant sows fed with an unprotected electronic sow feeder (fitmix). J. Appl. Anim. Welf. Sci. 11:319–336. 10.1080/10888700802329939 18821401

[txag049-B6] Cheng C. , Wu X., Zhang X., Zhang X., Peng J. 2019. Obesity of sows at late pregnancy aggravates metabolic disorder of perinatal sows and affects performance and intestinal health of piglets. Animals. 10:49. 10.3390/ani10010049 31881697 PMC7023453

[txag049-B7] Cozannet P et al 2018. Reducing BW loss during lactation in sows: a meta-analysis on the use of a nonstarch polysaccharide-hydrolyzing enzyme supplement. J. Anim. Sci. 96:2777–2788. 10.1093/jas/sky045 29471398 PMC6095383

[txag049-B8] Dourmad J. Y. , Étienne M., Noblet J., Causeur D. 1997. Prédiction de la composition chimique des truies reproductrices à partir du poids vif et de l’épaisseur de lard dorsal: application à la définition des besoins énergétiques. Journal de Recherches Porcines en France. 29:255–262.

[txag049-B9] Feldpausch J. A. et al 2019. Birth weight threshold for identifying piglets at risk for preweaning mortality. Transl. Anim. Sci. 3:633–640. 10.1093/tas/txz076 32704833 PMC7200817

[txag049-B10] Gerritsen , Bikker R. P., van der Aar P. 2010. Glucose metabolism in reproductive sows. In: Dynamics in animal nutrition. Brill | Wageningen Academic. p. 99–112.

[txag049-B11] Gonçalves M. A. D. et al 2016. Effects of amino acids and energy intake during late gestation of high-performing gilts and sows on litter and reproductive performance under commercial conditions1,2. J. Anim. Sci. 94:1993–2003. 10.2527/jas.2015-0087 27285697

[txag049-B12] Gourley K. M. et al 2020. Effects of timing and size of meals prior to farrowing on sow and litter performance. Transl. Anim. Sci. 4:txaa066. 10.1093/tas/txaa066 32705061 PMC7281871

[txag049-B13] Ha S. H. et al 2024. Correlation between reproductive performance and sow body weight change during gestation. J. Anim. Sci. Technol. 66:543–554. 10.5187/jast.2023.e105 38975586 PMC11222117

[txag049-B14] Holt J. P. , Johnston L. J., Baidoo S. K., Shurson G. C. 2006. Effects of a high-fiber diet and frequent feeding on behavior, reproductive performance, and nutrient digestibility in gestating sows1,2. J. Anim. Sci. 84:946–955. 10.2527/2006.844946x 16543573

[txag049-B15] Huber L. 2025. Review: Feeding strategies to meet the dynamic lysine and energy requirements of gestating and lactating sows. Animal. 101574:101574. 10.1016/j.animal.2025.101574 40617771

[txag049-B16] Jung S.-W et al 2024. Effect of feeding frequency on reproductive performances and stress responses in gestating sows. J. Anim. Sci. Technol. 66:135–144. 10.5187/jast.2023.e42 38618032 PMC11007455

[txag049-B29969651] Knap P. W et al 2023. Genetic and phenotypic time trends of litter size, piglet mortality, and birth weight in pigs. Front. Anim. Sci. 4:1218175. 10.3389/fanim.2023.1218175

[txag049-B17] Liu Z. H. et al 2020. Effect of increasing feed intake during late gestation on piglet performance at parturition in commercial production enterprises. Anim. Reprod. Sci. 218:106477. 10.1016/j.anireprosci.2020.106477 32507257

[txag049-B18] Mallmann A. L. et al 2019. Impact of feed intake during late gestation on piglet birth weight and reproductive performance: a dose-response study performed in gilts. J. Anim. Sci. 97:1262–1272. 10.1093/jas/skz017 30649395 PMC6396255

[txag049-B19] Manu H et al 2019a. Effects of time of feeding during gestation on sow’s performance1. J. Anim. Sci. 97:1234–1241. 10.1093/jas/skz006 30649344 PMC6396240

[txag049-B20] Manu H et al 2019b. Effect of feeding frequency and sow parity based on isocaloric intake during gestation on sow performance. J. Anim. Sci. 97:2154–2164. 10.1093/jas/skz099 30911756 PMC6488309

[txag049-B21] Manu H. , Lee S., Keyes M. C., Cairns J., Baidoo S. K. 2020. Behavioral and cortisol responses to feeding frequency in pregnant sows under isocaloric intake. J. Anim. Sci. 98 10.1093/jas/skaa226 PMC7431211 32681641

[txag049-B22] McClellan K. A. et al 2025. Feeding a nutrient enriched diet to late gestating sows across consecutive cycles improves micronutrient status, farrowing duration, and prolificacy. Transl. Anim. Sci. 9:txaf155. 10.1093/tas/txaf155 41439040 PMC12721376

[txag049-B23] Mehtab A et al 2026. A comprehensive review of bump-feeding strategies during late gestation in sows: nutritional and behavioral implications for farrowing performance and reproductive outcomes. Agriculture. 16:302. 10.3390/agriculture16030302

[txag049-B24] Monteiro M. S. et al 2025. The role of nutrition across production stages to improve sow longevity. Animals. 15:189. 10.3390/ani15020189 39858189 PMC11758652

[txag049-B25] Mosnier E et al 2010. Reduced feed intake of lactating primiparous sows is associated with increased insulin resistance during the peripartum period and is not modified through supplementation with dietary tryptophan1. J. Anim. Sci. 88:612–625. 10.2527/jas.2008-1768 19855001

[txag049-B26] National Research Council. 2012. Nutrient requirements of swine. 11th rev. ed. The National Academies Press, Washington, DC.

[txag049-B27] Oliviero R. A. et al 2020. Supplying sows energy on the expected day of farrowing improves farrowing kinetics and newborn piglet performance in the first 24 h after birth. Animal. 14: 2271–2276. 10.1017/S1751731120001317 32580812

[txag049-B270] Oliveira C. 2023. Offspring of hyper prolific sows: immunity, birthweight, and heterogeneous litters. Mol. Reprod. Dev. 90:580–584. 10.1002/mrd.2357235460115 35460115

[txag049-B28] Olsson A.-C. , Andersson M., Botermans J., Rantzer D., Svendsen J. 2011. Animal interaction and response to electronic sow feeding (ESF) in 3 different herds and effects of function settings to increase capacity. Livest. Sci. 137:268–272. 10.1016/j.livsci.2010.10.014

[txag049-B29] Peng X et al 2025. Analysis and prediction of backfat thickness in gestating sows using machine learning algorithms. Smart Agric. Technol. 11:100875. 10.1016/j.atech.2025.100875

[txag049-B30] Quiniou N. , Dagorn J., Gaudré D. 2002. Variation of piglets’ birth weight and consequences on subsequent performance. Livest. Prod. Sci. 78:63–70. 10.1016/S0301-6226(02)00181-1

[txag049-B31] Ren P. , Yang X. J., Kim J. S., Menon D., Baidoo S. K. 2017. Effect of different feeding levels during three short periods of gestation on sow and litter performance over two reproductive cycles. Anim. Reprod. Sci. 177:42–55. 10.1016/j.anireprosci.2016.12.005 28041653

[txag049-B32] Richard C. 2013. Analysis of sources of variation and relationships among sow productivity traits [MSc thesis]. Purdue University. https://docs.lib.purdue.edu/open_access_theses/21

[txag049-B33] Ruggeri R. , Bee G., Ollagnier C. 2025. Review: Intrauterine growth restriction, diagnosis and physiological characterisation in pigs. Animal. 19:101590. 10.1016/j.animal.2025.101590 40752266

[txag049-B34] Solomon T. P. J. , Chambers E. S., Jeukendrup A. E., Toogood A. A., Blannin A. K. 2008. The effect of feeding frequency on insulin and ghrelin responses in human subjects. Br. J. Nutr. 100:810–819. 10.1017/S000711450896757X 18394217

[txag049-B35] Theil P. K. , Farmer C., Feyera T. 2022. Review: Physiology and nutrition of late gestating and transition sows. J. Anim. Sci. 100 10.1093/jas/skac176 PMC9202569 35708593

[txag049-B36] Theil P. K. , Krogh U., Bruun T. S., Feyera T. 2023. Feeding the modern sow to sustain high productivity. Mol. Reprod. Dev. 90:517–532. 10.1002/mrd.23571 35451142

[txag049-B37] Tucker B. S. et al 2022. Increased feeding frequency prior to farrowing: effects on sow performance. Transl. Anim. Sci. 6:txac062. 10.1093/tas/txac062 35673542 PMC9168070

[txag049-B38] van den Bosch M. , Soede N., Kemp B., van den Brand H. 2023. Sow nutrition, uterine contractions, and placental blood flow during the peri-partum period and short-term effects on offspring: a review. Animals. 13:910. 10.3390/ani13050910 36899765 PMC10000096

[txag049-B39] Vargovic L. , Hermesch S., Athorn R. Z., Bunter K. L. 2021. Feed intake and feeding behavior traits for gestating sows recorded using electronic sow feeders. J. Anim. Sci. 99:skaa395. 10.1093/jas/skaa395 PMC7799585 33313717

[txag049-B40] Vodolazska D. , Feyera T., Lauridsen C. 2023. The impact of birth weight, birth order, birth asphyxia, and colostrum intake per se on growth and immunity of the suckling piglets. Sci. Rep. 13:8057. 10.1038/s41598-023-35277-3 37198433 PMC10192303

[txag049-B41] Wang T et al 2025. Effects of fiber concentrations and fermentation rates on reproductive performance, nutrient digestibility, immune response, and microbiota of lactating sows. J. Anim. Sci. 103:skaf110. 10.1093/jas/skaf110 PMC12199346 40290056

[txag049-B42] Wittmann M. 1986. Effect of frequency of feedings on reproductive performance of sows. Reports of the Research Centre for Animal Production and Nutrition (Hungary). 193.

[txag049-B43] Zhao Y , Kim S. W. 2020. Oxidative stress status and reproductive performance of sows during gestation and lactation under different thermal environments. Asian-Australas. J. Anim. Sci. 33:722–731. 10.5713/ajas.19.0334 32054225 PMC7206402

[txag049-B44] Zięcik A. , Kapelański W., Zaleska M., Riopérez J. 2002. Effect of diet composition and frequency of feeding on postprandial insulin level and ovarian follicular development in prepubertal pigs. J. Anim. Feed Sci. 11:471–483. 10.22358/jafs/67902/2002

