# Supplementary material for: Twice daily feeding during gestation improves maternal body reserves and fetal growth in sows under commercial conditions
Source: Transl Anim Sci. 2026 Apr 29;10:txag049. doi: 10.1093/tas/txag049 (PMC13186184; doi:10.1093/tas/txag049)
Supplement: txag049_Supplementary_Data [file txag049_supplementary_data.docx]

**Supplementary Table 1.** Sow performance and litter traits for parity × feeding frequency

| **Parameters** | **Parity 1-2** | | **Parity 3-5** | | **Parity ≥6** | |
| --- | --- | --- | --- | --- | --- | --- |
|  | Once | Twice | Once | Twice | Once | Twice |
| Numbers of sows | 67 | 62 | 70 | 84 | 19 | 36 |
| **ADFI (kg/d)** |  |  |  |  |  |  |
| d 1–35 | 2.66±0.39 | 2.64±0.40 | 2.74±0.38 | 2.72±0.39 | 2.73±0.42 | 2.71±0.43 |
| d 36–80 | 2.23±0.52 | 2.25±0.51 | 2.30±0.48 | 2.32±0.49 | 2.27±0.50 | 2.29±0.51 |
| d 81–110 | 2.79±0.61 | 2.81±0.60 | 2.87±0.58 | 2.89±0.59 | 2.84±0.63 | 2.86±0.64 |
| **BW (kg)** |  |  |  |  |  |  |
| d 1 | 184.0±22.1 | 186.2±23.2 | 196.9±25.1 | 199.1±26.3 | 204.4±28.8 | 206.6±29.8 |
| d 35 | 187.0±18.0 | 191.8±18.9 | 201.3±17.4 | 206.1±18.4 | 204.6±21.0 | 209.4±22.2 |
| d 80 | 192.9±21.5 | 201.2±22.5 | 212.2±21.8 | 220.5±22.8 | 215.0±24.5 | 223.3±25.5 |
| d 110 | 203.3±23.4 | 209.3±24.4 | 222.4±23.6 | 228.4±24.6 | 225.2±27.0 | 231.2±28.0 |
| BW change (d 1–110, kg) | 19.3±7.5 | 23.4±7.8 | 25.5±7.0 | 29.6±6.8 | 20.8±8.1 | 24.9±8.0 |
| **BF (mm)** |  |  |  |  |  |  |
| d 1 | 14.7±2.9 | 14.9±3.0 | 15.6±3.0 | 15.8±3.1 | 15.8±3.4 | 16.0±3.5 |
| d 35 | 14.9±2.5 | 15.2±2.6 | 16.0±2.6 | 16.3±2.7 | 15.9±3.0 | 16.2±3.1 |
| d 80 | 15.5±2.3 | 16.3±2.4 | 16.8±2.4 | 17.6±2.5 | 16.9±2.7 | 17.7±2.8 |
| d 110 | 15.3±2.5 | 16.2±2.6 | 16.7±2.6 | 17.6±2.7 | 16.8±2.9 | 17.7±3.0 |
| BF change (d 1–110, mm) | 0.6±1.3 | 1.3±1.3 | 1.1±1.3 | 1.8±1.3 | 1.0±1.5 | 1.7±1.5 |
| **Litter traits** |  |  |  |  |  |  |
| Total born | 19.1±5.3 | 19.4±5.5 | 22.6±4.2 | 22.9±4.4 | 21.6±4.1 | 21.9±4.3 |
| Live-born | 17.6±4.6 | 18.0±4.8 | 20.1±3.7 | 20.5±3.9 | 19.4±3.7 | 19.8±3.9 |
| Stillborn | 0.9±1.8 | 1.0±2.0 | 1.7±1.7 | 1.8±1.9 | 1.6±1.7 | 1.7±1.9 |
| Mummies | 0.6±1.2 | 0.4±1.2 | 0.8±0.9 | 0.6±0.9 | 0.6±0.7 | 0.4±0.7 |
| Piglet birth weight (kg) | 1.15±0.19 | 1.29±0.19 | 1.16±0.19 | 1.30±0.19 | 1.18±0.17 | 1.32±0.17 |
| Birth weight CV (%) | 20.3±3.9 | 19.6±3.9 | 21.8±3.7 | 21.1±3.7 | 22.2±4.3 | 21.5±4.3 |
| Farrowing duration (min) | 260±100 | 290±100 | 248±99 | 278±99 | 257±103 | 287±103 |

^1^ Values are presented as mean ± SD.
